# Is inhaled colistin beneficial in ventilator associated pneumonia or nosocomial pneumonia caused by *Acinetobacter baumannii?*

**DOI:** 10.1186/s12941-016-0123-7

**Published:** 2016-02-24

**Authors:** Tuna Demirdal, Ummu Sena Sari, Salih Atakan Nemli

**Affiliations:** Department of Infectious Disease, Faculty of Medicine, Ataturk Training and Research Hospital, Izmir Katip Celebi University, Basin sitesi, 35360 Izmir, Karabaglar Turkey

## Abstract

**Background:**

In the present study, our objective was to evaluate and compare the clinical and microbiological results in patients receiving systemic and systemic plus inhaled colistin therapy due to nosocomial pneumonia (NP) or ventilator associated pneumonia (VAP) caused by *Acinetobacter baumannii*.

**Methods:**

A retrospective matched case–control study was performed at the ICUs at Izmir Katip Celebi University Ataturk Training and Research Hospital from January 2013 to December 2014. Eighty patients who received only systemic colistin were matched 43 patients who received systemic colistin combined with inhaled therapy.

**Results:**

In 97.6 % of the patients colistin was co-administered with at least one additional antibiotic. The most frequently co-administered antibiotics were carbapenems (79.7 %). The patient groups did not differ significantly in terms of the non-colistin antibiotics used for treatment (p > 0.05). Acute renal injury was observed in 53.8 % and 48.8 % of the patients who received parenteral colistin or parenteral plus inhaler colistin, respectively (p = 0.603). There were no significant differences between the groups in terms of clinical success (p = 0.974), clinical failure (p = 0.291), or recurrence (p = 0.094). Only, a significantly higher partial clinical improvement rate was observed in the systemic colistin group (p = 0.009). No significant differences between the two groups in terms of eradication (p = 0.712), persistence (p = 0.470), or recurrence (p = 0.356) rates was observed. One-month mortality rate was similar in systemic (47.5 %) and systemic plus inhaled (53.5 %) treatment groups (p = 0.526).

**Conclusions:**

Our results suggest that combination of inhaled colistin with intravenous colistin had no additional therapeutic benefit in terms of clinical or microbiological outcomes.

## Backgrounds

Nosocomial pneumonia and ventilator associated pneumonia (VAP) represent the most common form of healthcare associates infections (HAIs) [1]. Commonly gram negative bacteria with potential multiple drug resistance (MDR) such as *Acinetobacter baumannii, Pseudomonas aeruginosa* and *Klebsiella pneumoniae* are commonly associated with nosocomial pneumonia, particularly in intensive care units (ICU). The mortality rate of nosocomial pneumonia may reach up to 70 %, with even higher mortality rates when the causative agent has MDR [2]. Recent evidence is indicative of a continuous increase in the detection rate of antibiotic resistance in these microorganisms [3].

Colistin represents an important therapeutic option in infections caused by MDR gram negative bacteria, although there is a certain level of clinical reluctance to its use due to low penetration in the lung parenchyma as well as the efficacy of intravenous colistin in patients with suspected pneumonia [4]. Inhalational use of colistin prevents systemic side effects, while providing high concentrations in the airways represents a significant advantage [5]. Studies have suggested that the lung concentrations of colistin obtained following the inhalational route of administration may reach levels adequate to eradicate the susceptible *A. baumannii* strains [6]. Currently, inhaled colistin is widely and effectively used for the treatment of pulmonary exacerbations in cystic fibrosis patients colonized by *P. aeruginosa* [7]. On the other hand, experience on the use of colistin in pneumonia caused by other MDR gram negative bacteria is limited. In the present study, our objective was to retrospectively evaluate and compare the clinical and microbiological results in patients receiving systemic and systemic plus inhaled colistin therapy due to nosocomial pneumonia or (VAP) caused by *A. baumannii*.

## Methods

Patients admitted to ICU for a minimum duration of 48 h between January 2013 and December 2014 were included in this retrospective study if they received systemic treatment or systemic treatment in combination with inhaler colistin due to VAP or nosocomial pneumonia caused by *A. baumannii*. Patients whose length of ICU stays were shorter than 48 h were excluded as well as patients under 18 years of age. In addition to demographic data, duration of treatment, concomitant antibiotics, presence of acute renal failure, and clinical and microbiological outcomes were assessed. Systemic colistin dose was administered via intravenous route as 150 mg colistin base activity q12 h and inhaled colistin dose was administered via nebulizer 75 mg colistin base activity q12 h. Diagnosis of nephrotoxicity was based on RIFLE criteria [8]. A ≥1.5-fold increase in creatinine levels from baseline to treatment end was considered to represent acute renal injury. Strains isolated were identified using conventional methods. Definitive typing of the strains was performed using an automated BD Phoenix system (Becton Dickinson, USA) with antibiotic susceptibility testing. Results were analyzed using SPSS v22 software package.

For a diagnosis of pneumonia, at least two of the following supportive clinical signs were required in the presence of new or progressive pulmonary infiltrates in radiographic assessments: a body temperature >38 °C or <35.5 °C, leukocyte count >12,000 cells/mm^3^ or <4000 cells/mm^3^, purulent bronchial secretions, or reduced oxygenation [9]. Nosocomial pneumonia was defined as the one that was not in incubation period at admission and that developed >48 h after admission, while VAP was defined as the one that was not in the incubation period before the initiation of mechanical ventilation and that developed >48 h after intubation. A microbiological diagnosis was ascertained by growth of >10^4^ CFU/ml in the bronchial or bronchoalveolar (BAL) fluid [10].

Clinical outcome was categorized as clinical success (resolution of the infection related signs and symptoms with colistin treatment), partial clinical improvement (partial improvement in the symptoms and signs of infection), clinical failure (persistence or worsening of the symptoms and signs of infection despite colistin administered at appropriate dose), recurrence (occurrence of a new infection episode at least 48 h after the initial clinical improvement with colistin). Microbiological results were categorized as eradication (eradication of the causative organism in samples after treatment), persistence (persistence of the organism in clinical samples irrespective of the clinical status of the patient), recurrence (re-identification of the causative pathogen in clinical samples after initial eradication), colonization (persistence or re-growth of the organism in clinical samples without any signs and symptoms of the infection). One-month mortality was defined as death occurring within 30 days after the isolation of the causative organism.

## Results

Of the 123 patients included, 40 (32.5 %) were female, and 83 (67.5 %) were male, with a mean age of 64.15 ± 17.75 (18–95) years. There were 83 cases (67.5 %) with VAP and 40 cases (32.5 %) with nosocomial pneumonia. The indications for intensive care admission included acute neurological disorder in 38.2 %, respiratory failure in 26 % (n = 32), post-operative resuscitation in 15.4 % (n = 19), trauma in 8.9 % (n = 11), septic shock in 5.7 % (n = 7), and cardiac arrest in 5.7 % (n = 7). Respiratory failure was significantly more common among those who received only systemic therapy (p = 0.025), while trauma was significantly more common in those who received systemic and inhaled therapy (p = 0.010). Other demographic characteristics were similar across the patients (Table 1). In 65 % of the cases (n = 80) only systemic colistin was administered, while it was co-administered with inhaled therapy in 35 % (n = 43). In 97.6 % of the patients (n = 120) colistin was co-administered with at least one additional antibiotic. The most frequently co-administered antibiotics were carbapenems (n = 98, 79.7 %), cefoperazone-sublactam (n = 15, 12.2 %), followed by tigecycline (n = 7, 5.7 %), sulbactam (n = 4, 3.3 %), and ceftazidim (n = 1, 0.8 %). Three patients (2.4 %) received carbapenem and tigecycline in conjunction with colistin, two patients (1.6 %) received carbapenem and cefoperazone-sulbactam with colistin, 1 patient (0.8 %) received tigecycline and sulbactam with colistin. The patient groups did not differ significantly in terms of the non-colistin antibiotics used for treatment (p > 0.05).

**Table 1:** Demographics of the patients received systemic, inhaler and systemic colistin therapy

	PE colistin (n=80)	PE and inh. colistin	p
	n (%)	n (%)	
Age ± SD	62.80 ± 18.80	66.67 ± 15.49	0.290
Gender (M/F)	50/30	33/10	0.109
Cause of admission			0.386
Respiratory failure	26 (32.5)	6 (14)	0.025
Septic shock	3 (3.8)	4 (9.3)	0.205
Trauma	7 (8.8)	4 (9.3)	0.010
Post-op resuscitation	10 (12.5)	9 (20.9)	0.217
Acute neurological disorder	29 (36.3)	18 (41.9)	0.541
Cardiac arrest	5 (6.3)	2 (4.7)	0.715
Duration of ICU stay ± SD	57.68 ± 56.99	47.91 ± 47.02	0.514
Duration of colistin therapy ± SD	11.21 ± 6.714	11.23 ± 6.023	0.708
Infection			
HKP	25 (31.3)	15 (34.9)	0.681
VIP	55 (68.7)	28 (65.1)	0.681
Creatinine at baseline ± SD	0.82 ± 0.56	1.20 ± 0.89	0.009
Creatinine at end-of-treatment ± SD	1.65 ± 1.08	1.86 ± 1.22	0.469
Nephrotoxic drug use	43 (53.8)	23 (53.5)	0.978
Underlying disorders			
Cerebrovascular event	17 (21.3)	11 (25.6)	0.584
COPD	6 (7.5)	3 (7)	0.915
CAD	28 (35)	22 (51.2)	0.081
Diabetes mellitus	19 (23.8)	10 (23.3)	0.950
CRF	5 (6.3)	3 (7)	0.876
Concomitant antibiotics			
Carbapenem	62 (77.5)	36 (83.7)	0.413
Cefoperazon sulbactam	9 (11.3)	6 (14)	0.662
Tigecycline	5 (6.3)	2 (4.7)	0.715
Sulbactam	2 (2.5)	2 (4.7)	0.521
Ceftazidime	1 (1.3)	0 (0)	0.461

*PE* parenteral; *Inh* inhaled use

The average duration of intensive unit care was 63.1 ± 51.67 days (11–287), and the average duration of colistin therapy was 11.2 ± 6.45 days (3–32).

The average baseline and end-of-treatment creatinine levels in patients who received only systemic or systemic plus inhaler colistin therapy were 0.82 ± 0.56/1.20 ± 0.89; 1.65 ± 1.08/1.86 ± 1.22 mg/dl, respectively. In those who received systemic therapy, the baseline creatinine levels were significantly lower as compared to the other group (p = 0.009). Fifty-two percent (n = 64) of the patients developed acute renal injury. Treatment was discontinued in 22 patients (22 %) due to nephrotoxicity, and 5 patients (4.1 %) required dialysis. Acute renal injury was observed in 53.8 % (n = 43) and 48.8 % (n = 21) of the patients who received parenteral colistin or parenteral plus inhaler colistin, respectively, with no significant difference between the groups (p = 0.603). In addition to colistin, use of other nephrotoxic agents such as aminoglycosides or glycopeptides was also examined. Of the study subjects, 53.7 % (n = 66) had a history of the use of another nephrotoxic agents. While 54.5 % (n = 36) of those who received another nephrotoxic agent developed acute renal injury, this figure was 49.1 % (n = 28) in those with no use of these agents, and the difference was not significant between the two groups (p = 0.548).

Table 2 shows the clinical and microbiological outcomes as well as the one-month mortality rates in the treatment groups. Clinical success, partial improvement, clinical failure and recurrence were observed in 37.4 % (n = 46), 13 % (n = 57), 44.7 % (n = 55), and 4.9 % (n = 55), respectively. Clinical success was observed in 37.5 % (n = 30) of the patients who received parenteral colistin only, while this figure was 37.2 % (n = 16) among those who received parenteral plus inhaler colistin, with no significant differences between the groups in terms of clinical success (p = 0.974), clinical failure (p = 0.291), or recurrence (p = 0.094). Only, a significantly higher partial clinical improvement rate was observed in the systemic colistin group (p = 0.009).

**Table 2:** Clinical and microbiological outcome and mortality in patients receiving systemic or systemic + inhaled colistin therapy

	PE colistin	PE and inh. colistin	p
	n (%)	n (%)	
Clinical outcome			
Clinical success	30 (37.5)	16 (37.2)	0.974
Partial clinical improvement	15 (18.8)	1 (2.3)	0.009
Clinical failure	33 (41.3)	22 (51.2)	0.291
Recurrence	2 (2.5)	4 (9.3)	0.094
Microbiological outcome			
Eradication	40 (50)	20 (46.5)	0.712
Persistence	30 (37.5)	19 (44.2)	0.470
Recurrence	4 (5)	4 (9.3)	0.356
Colonization	6 (7.5)	0 (0)	0.062
Mortality	38 (47.5)	23 (53.5)	0.526
Side effects			
Nephrotoxicity	43 (53.8)	21 (48.8)	0.603
Neurotoxicty	0	0	
Local side effects	0	0	

*PE* parenteral; *Inh* inhaled

After the completion of colistin therapy, eradication, persistence, recurrence, or colonization were determined microbiologically in 48.8 % (n = 60), 39.8 % (n = 49), 6.5 % (n = 8), and 4.9 % (n = 6) of the patients, respectively. In those who received only parenteral colistin, the rate of microbiologic eradication was 50 % (n = 40), while this figure was 46.5 % in those who received parenteral and inhaled colistin (n = 20). No significant differences between the two groups in terms of eradication (p = 0.712), persistence (p = 0.470), or recurrence (p = 0.356) rates. There were no cases of colonization in the group that received inhaled colistin, while the difference with the group that received systemic therapy only was insignificant (p = 0.062).

One-month mortality rate after colistin therapy was 49.6 % (n = 61). This figure in those who received parenteral colistin or parenteral plus inhaled colistin was 47.5 % (n = 38) and 53.5 % (n = 23), with no significant difference (p = 0.526).

## Discussion

Despite excellent bactericidal activity of colistin against majority of gram-negative organisms, it’s ineffective against gram-positive bacteria and anaerobes other than *Prevotella* spp., and *Fusobacterium* spp. [11]. Although it has a wide-spectrum of activity, its use has been limited to problematic organisms such as *P. aeruginosa* and *A. baumannii* due to its side effects [4]. In the collaborative guidelines issued by the American Thoracic Society (ATS) and Infectious Diseases Society of America (IDSA), it is recommended as a last resort for the treatment of infections caused by MDR-negative gram negative organisms, and particularly for VAP developing in critically ill patients [10].

Ventilator associated pneumonia (VAP) is one of the most common types of HAIs, representing approximately 15 % of all HAIs [12] and being associated with the highest morbidity and mortality rates among infections in the intensive care unit. It accounts for nearly 50 % of the total antibiotic use in the ICU. The incidence of hard-to-treat organisms such as *A. baumannii*, *P. aeruginosa*, and carbapenem-resistant *Enterobacteriaceae* spp. is continuously increasing, with resistance reported against all antibiotics including colistin in some centers [13].

MDR gram negative bacterial infections are increasingly more frequently observed in intensive care units. Considering the limited therapeutic options and high mortality of these infections, the benefits that could be obtained through inhaled colistin use may be important. Theoretically, inhaled route of administration may allow direct access of the colistin to the site of infection and may allow prevention of renal or neurological side effects that may occur during systemic use [14]. Inhaled colistin is generally well tolerated, with rare cases of bronchoconstriction [15], and no increase in the incidence of serious systemic side effects such as nephrotoxicity have been reported with inhaled colistin therapy [14, 16–19]. Similarly, inhaled colistin was well tolerated in our study, with no systemic or local side effects after its use. These results suggest that inhaled colistin therapy may be a safe therapeutic option in terms of side effects.

Many studies have reported on the efficacy of colistin, an important therapeutic option, on MDR gram-negative bacteria [20]. However, one of the most common side effects of colistin therapy is nephrotoxicity, which is particularly more common in patients with high baseline creatinine at the initiation of treatment. On the other hand, the reported frequency and severity of nephrotoxicity is lower as compared to the figures reported in 1970s [21]. Tumbarello et al. [22]. reported that 22 % of the patients had nephrotoxicity during inhaled therapy, while this figure was 25 % in inhaled plus systemic therapy, with no significant differences (p = 0.62). Also, these authors suggested that the development of nephrotoxicity was a risk factor for treatment failure. Kalin et al. [23] showed an increased incidence of nephrotoxicity with high dose systemic colistin as compared to normal or lower doses in VAP patients, while the differences were again insignificant. Nephrotoxicity occurred at a higher frequency among patients receiving inhaled colistin, with no significant difference with other groups. The incidence of nephrotoxicity reported by Kofteridis et al. [14] and Rattanaumpawan et al. [17] were 19 % and 25.5 %, respectively. In our study, acute renal injury was assessed using the RIFLE criteria and 64 patients (52 %) were found to have acute renal injury. In 27 patients (22 %) nephrotoxicity resulted in the discontinuation of the treatment, and 5 patients (4.1 %) required hemodialysis. There were no significant differences in acute renal injury between patients who received systemic colistin and those who received combination of systemic and inhaled colistin (p = 0.603). A comparison between patients with or without a history of the use of another nephrotoxic agent revealed no difference in the risk of developing acute renal injury (p = 0.548), suggesting that the nephrotoxic effects observed are most likely due to colistin. However, the observed rate of nephrotoxicity in our study is higher than those reported previously, which might have been due to the differences between studies with respect to the definition used for nephrotoxicity.

Despite a number of observational studies on the effect of inhaled colistin on clinical outcomes, prospective case–control studies examining patients who did or did not receive inhaled colistin are non-existent [24]. The benefits of inhaled colistin have been subject to controversy, with some studies reporting beneficial effects, and others proposing no such benefits [14, 16, 25]. Kofteridis et al. [14] found no additional therapeutic benefits from adding inhaled colistin to systemic colistin in VAP caused by MDR gram-negative bacteria. Tumbarello et al. [22] observed significantly higher rates of clinical improvement in VAP caused by *A. baumannii*, *P. aeruginosa*, or *K. pneumonia* with inhaled colistin therapy. In another study examining patients with VAP caused by MDR *A. baumannii* strains, lower rates of clinical response was found among patients receiving inhaled therapy as compared to patients who received intravenous therapy only, although the difference was not statistically significant [23].

In a 2015 meta-analysis of inhaled colistin therapy in VAP patients, significant improvements in clinical outcome, microbiological eradication, and infection-related mortality rates were found when inhaled colistin was added to systemic colistin [26]. Our results did not show any difference between parenteral plus inhaled colistin therapy and parenteral colistin alone with regard to complete clinical response (p = 0.974). This result may be related with the fact that the clinical severity of the patients was not assessed in our study.

Microbiological cure could be achieved in 48.8 % of the patients who received systemic and inhaled colistin, with no differences between systemic colistin alone and combined inhaled and systemic colistin in this respect (p = 0.712). Hsieh et al. [27] reported a microbiological success rate of 75 % among patients colonized with *A. baumannii* or diagnosed with pneumonia, while the figure found by Kofteridis et al. [14] in patients with VAP was 45 %. In patients with VAP due to *A. baumannii*, the bacterial eradication rates with intravenous colistin treatment and inhaled plus systemic colistin treatment were 69 % and 76 %, respectively [23]. In the study by Rattanaumpawan et al. [17] addition of inhaled colistin to intravenous colistin a significant increase in microbiological cure rate was found (p = 0.03). Similarly Kuo et al. [19] found a positive effect of inhaled colistin in terms of microbiological success, and identified inhaled colistin as an independent predictor for eradication. On the other hand, Tumbarello et al. [22], in their study involving VAP patients, claimed that use of inhaled colistin had no meaningful effect on microbiological eradication. Similarly, our results showed no additional benefit from inhaled colistin. Use of inhaled colistin was associated with the absence of colonization, although the difference was not significant (p = 0.062). In several previous studies, the inoculum burden of a specific micro-organism was shown to represent an important determinant of eradication [28]. The fact that the inoculum burden was not assessed in our study may have had an effect on our results.

Microorganisms associated with highest mortality in VAP patients include *A. baumannii*, *P. aeruginosa*, and methicillin-resistant *S. aureus* [10], placing an extra significance on the investigation of an additional therapeutic benefit of inhaled colistin in these patients.

In our study, the one-month mortality was 61.4 %, with no significant difference between inhaled colistin alone and inhaled plus systemic colistin in terms of mortality (p = 0.942). In the study by Korbila et al. [18] involving VAP patients, mortality rate with inhaled colistin and combined systemic and inhaled colistin groups were 39.7 and 44.2 %, respectively. Again the overall mortality in VAP caused by MDR gram negative organisms in the study by Michalopoulos et al. [16] was 25 %, while the VAP-associated mortality was 16.7 %, among patients receiving inhaled colistin. In previous studies, mortality rates were comparable between inhaled colistin and intravenous plus inhaled colistin, with no significant differences [14, 18, 22, 29]. In line with these previous reports, we did not find any significant effect of inhaled treatment on mortality (p = 0.526).

In nosocomial pneumonia due to multi-drug resistant gram-negative bacteria, inhaled colistin therapy represents a promising approach owing to the side effects systemic colistin treatment. Our results suggest that combination of inhaled colistin with intravenous colistin and/or non-colistin antibiotics had no additional therapeutic benefit in terms of clinical or microbiological outcomes, with no additional mortality benefit. However due to the presence of certain limitations such as the retrospective nature of our study and absence of the assessment of the disease severity, further prospective-controlled studies are warranted to minimize the effect of such confounding factors.
